# Supplementary material for: Measures of possible allostatic load in comorbid cocaine and alcohol use disorder: Brain white matter integrity, telomere length, and anti-saccade performance
Source: PLoS One. 2019 Jan 9;14(1):e0199729. doi: 10.1371/journal.pone.0199729 (PMC6326479; doi:10.1371/journal.pone.0199729)
Supplement: S1 File — Details for DTI methods, metrics, and analyses. (PDF) [file pone.0199729.s001.pdf]

S1 File. Details for DTI methods, metrics, and analyses.

DTI imaging techniques allow for the visualization of white matter (WM) fibers by quantifying the diffusive properties of water within tissue. The metrics typically used to describe diffusivity are axial, radial, and mean diffusivity (AD, RD, and MD, respectively), as well as fractional anisotropy (FA) values. AD measures diffusivity running along an axon while RD measures the diffusion perpendicular to an axon. Thus, high AD values can be interpreted as a sign of axonal integrity, while low AD values have been implicated in axonal degeneration [1]. Conversely, high RD values suggest possible demyelination [2] and are commonly associated with varying pathologies, such as multiple sclerosis [3], Alzheimer's [4], and schizophrenia [5]. MD is a weighted average of AD and RD, providing an inverse indicator of membrane density. FA is the weighted ratio of AD to RD, is sensitive to microstructural changes in WM, and thus serves as a summary metric of WM integrity, with higher FA values associated with healthy WM.

For the present study, the DTI scanning parameters were: FOV=256 mm; voxel size at acquisition =2 x 2 x 2, interpolated to 1x1x2mm; matrix =256 x 256 x 70; b=1000 s/mm/mm; TE=65 ms; TR=9100 ms; Encoding = alternating polarity Icosa42. Diffusion weighted images were processed using the FMRIB Diffusion Toolbox (FDT) within FSL [6, 7]. FA, MD, RD, and AD maps for each subject were generated after correcting for eddy current distortions, creating a brain mask by removing all non-brain tissue using the brain extraction tool (BET), and fitting a diffusion tensor model to raw diffusion data (DTIFIT). The Tract-Based Spatial Statistics (TBSS) method was then used for voxel-wise whole brain analysis. All FA maps were transformed into MNI152 standard space by nonlinearly registering each map to a standard template (FMRIB58_FA) using a non-linear registration method (FNIRT) as implemented in FSL [8]. The FMRIB58_FA template is a high-resolution brain average of 58 healthy subjects of both genders.

After registration, a mean FA skeleton was automatically generated from a mean FA map by tracing the center of common fiber bundles across all subjects. The mean FA skeleton was thresholded at an FA value of 0.2. An FA skeleton thresholded at 0.3 did not result in significantly different results. Each subject's FA map was projected onto the corresponding FA skeleton allowing for voxelwise analysis across subjects using a permutation-based non-parametric statistical method - (RANDOMISE) [9]. The Threshold-Free Cluster Enhancement (TFCE) option was selected and 5000 permutations were used.

References

1. Song S, Sun S, Ju W, Lin S, Cross AH, Neufeld. Diffusion tensor imaging detects and differentiates axon and myelin degeneration in mouse optic nerve after retinal ischemia. NeuroImage 2003; 20: 1714-1722.
2. Song S, Sun S, Ramsbottom MJ, Chang C, Russell J, Cross AH. Dysmyelination revealed through MRI as increased radial (but unchanged axial) diffusion of water. NeuroImage 2002; 17: 1429-1436.
3. Sbardella E, Tona F, Petsas N, Pantano P. DTI Measurements in Multiple Sclerosis: Evaluation of brain damage and clinical implications. Multiple Sclerosis International 2013; 2013: 1-11.
4. Nir TM, Jahanshad N, Villalon-Reina JE, Toga AW, Jack CR, Weiner MW, Thompson, PM. Effectiveness of regional DTI measures in distinguishing Alzheimer's disease, MCI, and normal aging. NeuroImage: Clinical 2013; 3: 180-195.
5. Lee S, Kubicki M, Asami T, Seidman LJ, Goldstein JM, et al. Extensive white matter abnormalities in patients with first-episode schizophrenia: A diffusion tensor imaging (DTI) study. Schizophrenia Research 2013; 143: 231-238.
6. Jenkinson M, Beckmann CF, Behrens TE, Woolrich MW, Smith SM. FSL. NeuroImage 2012; 62: 782-90.
7. http://www.fmrib.ox.ac.uk/fsl.
8. Andersson JLR, Jenkinson M, Smith S. Non-linear registration, aka spatial normalisation. FMRIB technical report 2010: TR07JA2.
9. Winkler AM, Ridgway GR, Webster MA, Smith SM, Nichols TE. Permutation inference for the general linear model. NeuroImage 2014; 92: 381-397.
