# Supplementary material for: Measures of possible allostatic load in comorbid cocaine and alcohol use disorder: Brain white matter integrity, telomere length, and anti-saccade performance
Source: PLoS One. 2019 Jan 9;14(1):e0199729. doi: 10.1371/journal.pone.0199729 (PMC6326479; doi:10.1371/journal.pone.0199729)
Supplement: S2 File — Individual and group parameter estimates, and statistical and graphical convergence tests from the Hierarchical Bayesian Drift Diffusion Model (HDDM) output of analysis of the anti-saccade data, using the Python HDDM package. For further details, see (1) manuscript text; (2) Wiecki, Sofer & Frank (2013). Frontiers in Neuroinformatics, 7, 1–10: doi:10.3389/fninf.2013.00014; and (3) http://ski.clps.brown.edu/hddm_docs/. (PDF) [file pone.0199729.s002.pdf]

S2 File. Individual and group parameter estimates, and statistical and graphical convergence tests from the Hierarchical Bayesian Drift Diffusion Model (HDDM) output of analysis of the anti-saccade data, using the Python HDDM package. For further details, see (1) manuscript text; (2) Wiecki, Sofer & Frank (2013). Frontiers in Neuroinformatics, 7, 1-10: doi: 10.3389/fninf.2013.00014; and (3) http://ski.clps.brown.edu/hddm_docs/.

```
In [8]: hddm.analyze.gelman_rubin(models)

Out[8]:
{'a(0)': 1.0000257966269643,
 'a(1)': 0.99997638535177957,
 'a_std': 1.0009976273111636,
 'a_subj(0).12933': 1.0002264614048706,
 'a_subj(0).12945': 1.0007116967177661,
 'a_subj(0).13067': 0.9999604119487896,
 'a_subj(0).13076': 1.0006784376765689,
 'a_subj(0).13086': 1.0014356222962164,
 'a_subj(0).13110': 1.0009270508729231,
 'a_subj(0).13153': 0.99993411264894483,
 'a_subj(0).13224': 1.0003653727680686,
 'a_subj(0).13260': 1.0000977165200449,
 'a_subj(0).13266': 1.0006841797826154,
 'a_subj(0).13267': 0.99984185964536132,
 'a_subj(0).13306': 0.99980527806999542,
 'a_subj(0).13312': 1.0025601635713079,
 'a_subj(0).13321': 0.9999541431204847,
 'a_subj(0).13322': 1.0000672209945176,
 'a_subj(0).13332': 0.99986691132946615,
 'a_subj(0).13340': 0.99992917872709086,
 'a_subj(0).13354': 0.99984025420964007,
 'a_subj(0).13374': 1.0003241749573655,
 'a_subj(0).13377': 1.0001383489797584,
 'a_subj(0).13382': 1.0008065706832519,
 'a_subj(0).13409': 1.0013758186268782,
 'a_subj(0).13445': 0.99988611181193099,
 'a_subj(0).13450': 1.0006302731109582,
 'a_subj(0).13456': 0.99992175613866607,
 'a_subj(0).13459': 0.99986307049275902,
 'a_subj(0).13479': 1.0002464465580694,
 'a_subj(0).13482': 0.99999638671451108,
 'a_subj(0).13483': 0.99983189130278927,
 'a_subj(0).13484': 1.0001787736246461,
 'a_subj(0).13485': 1.0002148164054674,
 'a_subj(0).13491': 1.0005278149373209,
 'a_subj(0).13514': 1.0005634786921742,
 'a_subj(0).13523': 1.0002173744219816,
 'a_subj(0).13566': 1.0000002338713128,
 'a_subj(0).13572': 1.0005165760030765,
 'a_subj(0).13574': 1.0004022591594277,
 'a_subj(1).13202': 0.99992161910758515,
```

```
 'a_subj(1).13206': 1.0002906420572928,
 'a_subj(1).13207': 1.0001831724358901,
 'a_subj(1).13213': 1.0001426371120623,
 'a_subj(1).13217': 1.0002797130370098,
 'a_subj(1).13224': 1.0000293759430761,
 'a_subj(1).13227': 1.0000060449121098,
 'a_subj(1).13232': 0.99996554628797996,
 'a_subj(1).13608': 1.0002897854188224,
 'a_subj(1).13619': 1.0003035529341004,
 'a_subj(1).13624': 1.0010892026936138,
 'a_subj(1).13636': 0.99993401369901869,
 'a_subj(1).13669': 1.0008451465047186,
 'a_subj(1).13735': 1.0001830941820158,
 'a_subj(1).13762': 1.0003926746159908,
 'a_subj(1).13770': 1.0001882551439762,
 'a_subj(1).13808': 0.9998701235012406,
 'a_subj(1).13958': 1.0000828772822192,
 'a_subj(1).14004': 1.0008804985966342,
 'a_subj(1).14014': 1.0002958630672172,
 'a_subj(1).14020': 0.99999794393130381,
 'a_subj(1).14027': 0.99997254057518226,

't': 1.0006609892687526,
 't_std': 1.0007269661952851,
 't_subj.12933': 1.0004195890538641,
 't_subj.12945': 1.0001713292868244,
 't_subj.13067': 0.99982351793147239,
 't_subj.13076': 1.0002301049160669,
 't_subj.13086': 1.0005517150996621,
 't_subj.13110': 1.0007575338463406,
 't_subj.13153': 1.0001309118752644,
 't_subj.13202': 1.0003429302140721,
 't_subj.13206': 1.0006225205180801,
 't_subj.13207': 0.99986595324772753,
 't_subj.13213': 1.0001953087691968,
 't_subj.13217': 1.0003031729848026,
 't_subj.13224': 1.0001294961887104,
 't_subj.13227': 1.0002039083011727,
 't_subj.13232': 0.9999948768483049,
 't_subj.13260': 1.000702920150236,
 't_subj.13266': 1.0005630343509084,
 't_subj.13267': 1.0000627229378194,
 't_subj.13306': 0.99983693556744191,
 't_subj.13312': 1.000692449032434,
 't_subj.13321': 0.99999069372391269,
 't_subj.13322': 1.0000081625227337,
 't_subj.13332': 0.99997488416933367,
 't_subj.13340': 0.99999302956894787,
 't_subj.13354': 1.0003605089213037,
 't_subj.13374': 1.0003642550577145,
```

```
't_subj.13377': 1.0000128481015951,
't_subj.13382': 1.0001375736232954,
't_subj.13409': 1.0012488530374797,
't_subj.13445': 1.0002185778513408,
't_subj.13450': 1.0006110703341577,
't_subj.13456': 1.0003610763057136,
't_subj.13459': 1.0006062411025107,
't_subj.13479': 1.0002730868932257,
't_subj.13482': 0.99986057420640806,
't_subj.13483': 1.0001745849106971,
't_subj.13484': 1.0006614011437747,
't_subj.13485': 1.0002037435641113,
't_subj.13491': 1.0001472012533359,
't_subj.13514': 1.0003451830003265,
't_subj.13523': 0.99998200077900712,
't_subj.13566': 1.0006608553303937,
't_subj.13572': 1.000863188071436,
't_subj.13574': 1.000088596983369,
't_subj.13608': 1.0002589321434185,
't_subj.13619': 1.0002523826788428,
't_subj.13624': 1.0004864475699025,
't_subj.13636': 0.9999413590159828,
't_subj.13669': 1.0003645338035108,
't_subj.13735': 1.001079117676493,
't_subj.13762': 1.0001410383474876,
't_subj.13770': 1.0006222304900696,
't_subj.13808': 1.0002774609354723,
't_subj.13958': 0.99991296411722286,
't_subj.14004': 1.000230882270001,
't_subj.14014': 1.0006348940465157,
't_subj.14020': 1.00000817502367,
't_subj.14027': 1.00041617577399,

'v(coke)': 0.99992797258123356,
'v(neutral)': 1.0001673631254278,
'v(shape)': 0.99998482719954007,
'v_std': 1.0007064769502048,
'v_subj(coke).12933': 0.99999088482094933,
'v_subj(coke).12945': 1.0000386065022373,
'v_subj(coke).13067': 0.99989787115859319,
'v_subj(coke).13076': 1.0000043710020701,
'v_subj(coke).13086': 1.0003410886152977,
'v_subj(coke).13110': 1.0001131395764498,
'v_subj(coke).13153': 1.0006259338832879,
'v_subj(coke).13202': 1.0003003903519776,
'v_subj(coke).13206': 0.9999424027007161,
'v_subj(coke).13207': 0.99998375590484234,
'v_subj(coke).13213': 1.0005419160075295,
'v_subj(coke).13217': 1.0001258873921117,
'v_subj(coke).13224': 1.0001802149921559,
```

'v_subj(coke).13227': 0.99984193404437927,
'v_subj(coke).13232': 0.99995648099281731,
'v_subj(coke).13260': 1.0000595132987931,
'v_subj(coke).13266': 1.0007701640830602,
'v_subj(coke).13267': 1.0001060243117788,
'v_subj(coke).13306': 0.99989614729369691,
'v_subj(coke).13312': 1.0005834636665882,
'v_subj(coke).13321': 1.0001851998573952,
'v_subj(coke).13322': 0.99987021646372365,
'v_subj(coke).13332': 0.99991751852921973,
'v_subj(coke).13340': 1.0002166736804463,
'v_subj(coke).13354': 0.9999050718603919,
'v_subj(coke).13374': 1.0003225054032883,
'v_subj(coke).13377': 0.9999541381706436,
'v_subj(coke).13382': 1.0002592082875645,
'v_subj(coke).13409': 1.0004113994630213,
'v_subj(coke).13445': 0.99986710630820419,
'v_subj(coke).13450': 0.99996499317341581,
'v_subj(coke).13456': 0.99990712090977685,
'v_subj(coke).13459': 1.0000949487026483,
'v_subj(coke).13479': 1.0000319553518537,
'v_subj(coke).13482': 1.0002454547437816,
'v_subj(coke).13483': 1.0005211319912828,
'v_subj(coke).13484': 1.000042958930101,
'v_subj(coke).13485': 1.0000465731855328,
'v_subj(coke).13491': 1.0001452477374957,
'v_subj(coke).13514': 0.99992864863694741,
'v_subj(coke).13523': 1.0000636339554259,
'v_subj(coke).13566': 0.99983972708601609,
'v_subj(coke).13572': 1.0001841632968969,
'v_subj(coke).13574': 1.0002673301107807,
'v_subj(coke).13608': 1.0002510431907274,
'v_subj(coke).13619': 1.0000494732041696,
'v_subj(coke).13624': 0.99996038602873694,
'v_subj(coke).13636': 0.9998855474978311,
'v_subj(coke).13669': 1.0000447245133002,
'v_subj(coke).13735': 1.0000707465453129,
'v_subj(coke).13762': 1.0001681460703491,
'v_subj(coke).13770': 0.99987503050376347,
'v_subj(coke).13808': 0.99985619706613382,
'v_subj(coke).13958': 1.0000196196511735,
'v_subj(coke).14004': 1.0001848563935452,
'v_subj(coke).14014': 1.0007649370731484,
'v_subj(coke).14020': 1.0004187419895054,
'v_subj(coke).14027': 1.0000407939278186,
'v_subj(neutral).12933': 0.99991303325701031,
'v_subj(neutral).12945': 1.0000224350606151,
'v_subj(neutral).13067': 0.99988413535174314,
'v_subj(neutral).13076': 1.0003682767189688,
'v_subj(neutral).13086': 0.99989475963501417,

'v_subj(neutral).13110': 0.99989450934643165,
'v_subj(neutral).13153': 0.99985931638531189,
'v_subj(neutral).13202': 1.0000801754690141,
'v_subj(neutral).13206': 0.99998419709407393,
'v_subj(neutral).13207': 1.0000807988608451,
'v_subj(neutral).13213': 1.0002497150871015,
'v_subj(neutral).13217': 0.99986642895570121,
'v_subj(neutral).13224': 0.99993186576846649,
'v_subj(neutral).13227': 1.0002719481602038,
'v_subj(neutral).13232': 0.99997849199672018,
'v_subj(neutral).13260': 1.0001570482027424,
'v_subj(neutral).13266': 1.0002077187888747,
'v_subj(neutral).13267': 1.0002045225869205,
'v_subj(neutral).13306': 0.99990528453796956,
'v_subj(neutral).13312': 1.0008250693256444,
'v_subj(neutral).13321': 1.0001261710354692,
'v_subj(neutral).13322': 0.99991991800757418,
'v_subj(neutral).13332': 0.99990335566713939,
'v_subj(neutral).13340': 0.99995100534181014,
'v_subj(neutral).13354': 0.99998436928332357,
'v_subj(neutral).13374': 0.99989072707871207,
'v_subj(neutral).13377': 1.0000077071910809,
'v_subj(neutral).13382': 0.99997556899340523,
'v_subj(neutral).13409': 1.0001758688842297,
'v_subj(neutral).13445': 1.0001687780613833,
'v_subj(neutral).13450': 1.0001129689607451,
'v_subj(neutral).13456': 0.99996449154307254,
'v_subj(neutral).13459': 0.99991487153266723,
'v_subj(neutral).13479': 0.99984390949320412,
'v_subj(neutral).13482': 1.0000230175994511,
'v_subj(neutral).13483': 1.0000910840583004,
'v_subj(neutral).13484': 0.99992482312021935,
'v_subj(neutral).13485': 1.000153905510702,
'v_subj(neutral).13491': 1.0000065175635386,
'v_subj(neutral).13514': 1.0000774303730449,
'v_subj(neutral).13523': 0.9998898572864805,
'v_subj(neutral).13566': 0.99995032241551407,
'v_subj(neutral).13572': 0.99984051334011759,
'v_subj(neutral).13574': 0.9999290912621096,
'v_subj(neutral).13608': 1.0003264841559985,
'v_subj(neutral).13619': 1.0000042229842012,
'v_subj(neutral).13624': 1.0000869278882063,
'v_subj(neutral).13636': 0.99990683553343751,
'v_subj(neutral).13669': 1.0001349823562327,
'v_subj(neutral).13735': 0.99982711608295094,
'v_subj(neutral).13762': 1.0001638372941961,
'v_subj(neutral).13770': 0.99992987592502391,
'v_subj(neutral).13808': 1.0000300727176059,
'v_subj(neutral).13958': 1.0001825770238582,
'v_subj(neutral).14004': 1.0000871926018082,

'v_subj(neutral).14014': 0.99983136738539002,
'v_subj(neutral).14020': 1.0001962871879997,
'v_subj(neutral).14027': 1.0000291543597188,
'v_subj(shape).12933': 1.0000208402898487,
'v_subj(shape).12945': 1.0008110502430001,
'v_subj(shape).13067': 0.99993530293167276,
'v_subj(shape).13076': 1.0005506094889032,
'v_subj(shape).13086': 1.0004728120416233,
'v_subj(shape).13110': 1.0006313628299017,
'v_subj(shape).13153': 0.99986279444310822,
'v_subj(shape).13202': 1.0000628887392899,
'v_subj(shape).13206': 0.99986464138364839,
'v_subj(shape).13207': 0.99987833599825926,
'v_subj(shape).13213': 1.0001703984483115,
'v_subj(shape).13217': 1.0001282350860723,
'v_subj(shape).13224': 1.0004162527776232,
'v_subj(shape).13227': 1.0004285212879902,
'v_subj(shape).13232': 1.0005506008700327,
'v_subj(shape).13260': 1.0001759719785797,
'v_subj(shape).13266': 1.0005626707594684,
'v_subj(shape).13267': 1.0000869698207748,
'v_subj(shape).13306': 1.0006390667626008,
'v_subj(shape).13312': 1.0006044361062019,
'v_subj(shape).13321': 0.99991418157891088,
'v_subj(shape).13322': 1.0000082547980931,
'v_subj(shape).13332': 1.0002867379991014,
'v_subj(shape).13340': 0.99998560358826816,
'v_subj(shape).13354': 0.9998497570466568,
'v_subj(shape).13374': 1.0000916097701931,
'v_subj(shape).13377': 1.0000033127359615,
'v_subj(shape).13382': 1.0003352558986611,
'v_subj(shape).13409': 1.0001399699480729,
'v_subj(shape).13445': 1.0003526847070667,
'v_subj(shape).13450': 1.0000314922416798,
'v_subj(shape).13456': 1.000098727412263,
'v_subj(shape).13459': 0.99987090628791753,
'v_subj(shape).13479': 0.99986919664786333,
'v_subj(shape).13482': 0.99999715270249523,
'v_subj(shape).13483': 0.99980901868644301,
'v_subj(shape).13484': 0.99990267295242041,
'v_subj(shape).13485': 0.9999073329904381,
'v_subj(shape).13491': 1.0002375123750125,
'v_subj(shape).13514': 1.0001051396809824,
'v_subj(shape).13523': 0.99993304633556157,
'v_subj(shape).13566': 1.0002248461119594,
'v_subj(shape).13572': 1.000012826368732,
'v_subj(shape).13574': 0.99982091344378221,
'v_subj(shape).13608': 0.99994891391074658,
'v_subj(shape).13619': 1.0000929992520868,
'v_subj(shape).13624': 1.000079353267372,

```
 'v_subj(shape).13636': 1.0003378377623635,
 'v_subj(shape).13669': 0.99998689445573608,
 'v_subj(shape).13735': 1.000212168631512,
 'v_subj(shape).13762': 1.0003099989967552,
 'v_subj(shape).13770': 0.99985692427171713,
 'v_subj(shape).13808': 0.99984627830076922,
 'v_subj(shape).13958': 0.99990935864514485,
 'v_subj(shape).14004': 1.0002571745661075,
 'v_subj(shape).14014': 1.0007772044341434,
 'v_subj(shape).14020': 1.0000569530933654,
 'v_subj(shape).14027': 0.99998018342582862}
```

```
In [9]: model1.print_stats()
```

```
Out[9]:
mean std 2.5q 25q 50q 75q 97.5q mc err
a(0) 3.8494 0.133412 3.58603 3.76013 3.84873 3.93678 4.11281 0.00250641
a(1) 3.3526 0.162365 3.0296 3.24791 3.35032 3.45618 3.68999 0.00237226
a_std 0.766796 0.0890889 0.614654 0.702642 0.760305 0.824614 0.958553 0.00228657
a_subj(0).12933 4.7313 0.399133 3.9987 4.45488 4.70579 4.98822 5.57369 0.00804165
a_subj(0).12945 3.38316 0.265977 2.89662 3.19612 3.37193 3.55885 3.94191 0.00571519
a_subj(0).13067 3.57735 0.219839 3.17745 3.42422 3.56611 3.71795 4.04041 0.00336089
a_subj(0).13076 4.25036 0.392482 3.54692 3.97653 4.22836 4.50749 5.06071 0.010451
a_subj(0).13086 3.195 0.268571 2.71172 3.00539 3.18346 3.36882 3.76618 0.00739362
a_subj(0).13110 3.28546 0.224466 2.86 3.13001 3.28013 3.43465 3.74243 0.00406335
a_subj(0).13153 3.16869 0.211599 2.78671 3.02006 3.15852 3.30598 3.60596 0.00434398
a_subj(0).13224 2.58423 0.168635 2.26569 2.46809 2.57953 2.69155 2.93534 0.00373258
a_subj(0).13260 3.37463 0.247685 2.91967 3.20301 3.36021 3.53399 3.89393 0.00523399
a_subj(0).13266 6.11425 0.547603 5.09149 5.7312 6.10645 6.47203 7.24212 0.0177471
a_subj(0).13267 4.01707 0.260532 3.53829 3.8344 4.00267 4.18673 4.56493 0.00466631
a_subj(0).13306 4.44147 0.288794 3.91647 4.24163 4.42661 4.62716 5.05073 0.00526
a_subj(0).13312 6.10115 0.570836 5.04691 5.70098 6.07934 6.47784 7.29712 0.0170708
a_subj(0).13321 4.25568 0.293155 3.73063 4.05329 4.24136 4.44454 4.87216 0.00560421
a_subj(0).13322 3.85159 0.225442 3.43599 3.69145 3.84389 4.0017 4.31189 0.00406896
a_subj(0).13332 3.77835 0.227567 3.35945 3.61875 3.76882 3.92368 4.25278 0.00469794
a_subj(0).13340 3.90848 0.287268 3.37967 3.70975 3.89811 4.10018 4.48803 0.00557436
a_subj(0).13354 3.8527 0.228757 3.43178 3.69626 3.8453 3.99737 4.32564 0.0040941
a_subj(0).13374 2.14918 0.125886 1.92169 2.06139 2.14291 2.23094 2.41408 0.00252715
a_subj(0).13377 4.09521 0.245956 3.649 3.9244 4.08408 4.25158 4.60718 0.00460402
a_subj(0).13382 4.00852 0.349253 3.36053 3.76089 3.99499 4.2379 4.73413 0.00881942
a_subj(0).13409 5.14316 0.495554 4.26302 4.7925 5.11436 5.46989 6.16632 0.0161751
a_subj(0).13445 3.19257 0.185616 2.85362 3.06577 3.18558 3.31121 3.59806 0.00331202
a_subj(0).13450 3.54494 0.297332 3.00984 3.33878 3.53043 3.73409 4.17396 0.00711675
a_subj(0).13456 4.41835 0.321042 3.83182 4.19784 4.40218 4.62144 5.10121 0.00595091
a_subj(0).13459 3.23533 0.196564 2.87163 3.09416 3.22715 3.36443 3.64145 0.00385022
a_subj(0).13479 3.88128 0.241542 3.44257 3.71639 3.86713 4.03315 4.39698 0.00411971
a_subj(0).13482 4.01927 0.284139 3.50345 3.8195 4.00759 4.20453 4.62152 0.00554942
a_subj(0).13483 3.10195 0.191987 2.74784 2.96955 3.09502 3.22743 3.50398 0.00396512
a_subj(0).13484 4.88746 0.349018 4.25007 4.64357 4.87337 5.11951 5.60863 0.00792106
a_subj(0).13485 3.79562 0.246663 3.34259 3.62421 3.78623 3.95633 4.30688 0.00438947
```

```
a_subj(0).13491 4.95489 0.399329 4.23626 4.66941 4.93837 5.22728 5.76302 0.00848451
a_subj(0).13514 3.87528 0.370165 3.2241 3.61313 3.85369 4.1046 4.68454 0.0111917
a_subj(0).13523 3.77719 0.25567 3.3149 3.60033 3.76391 3.94168 4.33602 0.00426024
a_subj(0).13566 4.08928 0.237488 3.65466 3.92307 4.0861 4.24055 4.58668 0.00417994
a_subj(0).13572 2.91541 0.261651 2.45064 2.73543 2.89747 3.0742 3.48693 0.00741049
a_subj(0).13574 3.83289 0.244327 3.38711 3.65829 3.82514 3.99363 4.34293 0.00450815
a_subj(1).13202 3.20917 0.19041 2.84782 3.08121 3.20533 3.33262 3.60494 0.00346851
a_subj(1).13206 3.13014 0.20591 2.74704 2.98913 3.117 3.26327 3.56289 0.00414898
a_subj(1).13207 3.68796 0.239546 3.25445 3.51863 3.67633 3.84494 4.18303 0.00419653
a_subj(1).13213 2.80656 0.162608 2.50149 2.69578 2.80315 2.91571 3.14111 0.00305857
a_subj(1).13217 3.68153 0.257332 3.19823 3.50607 3.67754 3.8494 4.22066 0.00523897
a_subj(1).13224 2.56033 0.165115 2.26171 2.44554 2.55223 2.66568 2.90555 0.00316807
a_subj(1).13227 4.14399 0.294738 3.6046 3.93446 4.13569 4.33058 4.74405 0.00594051
a_subj(1).13232 3.33943 0.19368 2.99019 3.20389 3.33293 3.46599 3.74291 0.0040119
a_subj(1).13608 3.15988 0.182703 2.83023 3.03104 3.15091 3.27563 3.53933 0.00335662
a_subj(1).13619 2.70192 0.152376 2.41958 2.5984 2.69516 2.80181 3.01831 0.00308187
a_subj(1).13624 3.98742 0.327158 3.40844 3.75885 3.97104 4.19332 4.67441 0.00803011
a_subj(1).13636 3.22835 0.186979 2.87958 3.10129 3.21718 3.34561 3.6192 0.00332427
a_subj(1).13669 2.27989 0.134959 2.03569 2.18779 2.27203 2.3659 2.55799 0.00258736
a_subj(1).13735 3.58655 0.214807 3.17783 3.44094 3.57603 3.72533 4.0285 0.00336267
a_subj(1).13762 3.88444 0.330556 3.29309 3.65769 3.86647 4.0924 4.6042 0.00870675
a_subj(1).13770 3.5392 0.194403 3.16989 3.40645 3.53393 3.6662 3.94518 0.0032087
a_subj(1).13808 3.50916 0.193911 3.15019 3.37753 3.50426 3.63234 3.9121 0.00359726
a_subj(1).13958 3.54706 0.150766 3.26459 3.44311 3.54483 3.64768 3.84682 0.00326684
a_subj(1).14004 3.87375 0.239434 3.43184 3.7052 3.8648 4.03638 4.36082 0.0040674
a_subj(1).14014 2.99822 0.178351 2.6676 2.87405 2.98969 3.11574 3.37143 0.00459408
a_subj(1).14020 3.34848 0.182012 3.01128 3.22424 3.34073 3.46796 3.72081 0.00357291
a_subj(1).14027 2.71613 0.15458 2.43651 2.60822 2.7087 2.81465 3.03927 0.00287497
v(coke) -0.281978 0.0581662 -0.394759 -0.321055 -0.280722 -0.242629 -0.167811 0.000984106
v(neutral) -0.311655 0.0599563 -0.428074 -0.351295 -0.31166 -0.270946 -0.196323 0.0010238
v(shape) -0.39394 0.0589299 -0.510095 -0.433631 -0.392621 -0.353699 -0.278781 0.000918467
v_std 0.420628 0.0271763 0.371855 0.401461 0.419027 0.438053 0.477538 0.000611551
v_subj(coke).12933 -0.519872 0.122111 -0.754592 -0.602914 -0.520399 -0.436317 -0.284243 0.00200144
v_subj(coke).12945 -0.624065 0.166096 -0.95061 -0.735891 -0.621837 -0.511181 -0.294603 0.00262959
v_subj(coke).13067 -0.214335 0.124579 -0.45266 -0.298436 -0.214766 -0.131261 0.033725 0.00175716
v_subj(coke).13076 -0.729383 0.156703 -1.02781 -0.835088 -0.729987 -0.622742 -0.421285 0.00275581
v_subj(coke).13086 -1.03219 0.189595 -1.41562 -1.15849 -1.02983 -0.906785 -0.659883 0.0034343
v_subj(coke).13110 -0.475938 0.147157 -0.766181 -0.57675 -0.475514 -0.377634 -0.189033 0.00220274
v_subj(coke).13153 -0.511079 0.14139 -0.795989 -0.602824 -0.509006 -0.414314 -0.241695 0.00233141
v_subj(coke).13202 0.155439 0.138347 -0.119815 0.0621145 0.152679 0.251638 0.424784 0.00178492
v_subj(coke).13206 0.164622 0.147392 -0.126299 0.067852 0.162216 0.264673 0.452509 0.00200149
v_subj(coke).13207 -0.388673 0.128574 -0.634518 -0.475109 -0.390919 -0.301953 -0.133101 0.00182178
v_subj(coke).13213 0.343701 0.15409 0.0428429 0.239475 0.345274 0.448915 0.648317 0.00209813
v_subj(coke).13217 -0.0242542 0.134404 -0.276896 -0.118065 -0.0233801 0.0667122 0.238485 0.00200105
v_subj(coke).13224 -0.392669 0.138178 -0.662718 -0.486529 -0.393399 -0.301066 -0.10769 0.00200202
v_subj(coke).13227 -0.263336 0.123067 -0.497235 -0.34894 -0.264913 -0.179374 -0.0216376 0.00160211
v_subj(coke).13232 0.0909073 0.131508 -0.164889 0.00330924 0.0913245 0.179893 0.34441 0.00198235
v_subj(coke).13260 -0.558788 0.153265 -0.857865 -0.662018 -0.559753 -0.454506 -0.259393 0.00226275
v_subj(coke).13266 -0.824424 0.128837 -1.07794 -0.909503 -0.824074 -0.737255 -0.571851 0.00319505
v_subj(coke).13267 -0.221539 0.118337 -0.448582 -0.3006 -0.221744 -0.142457 0.0137046 0.00161877
```

v_subj(coke).13306 -0.2428 0.105764 -0.450534 -0.315224 -0.244364 -0.173443 -0.0350058 0.00136213
v_subj(coke).13312 -0.60337 0.117164 -0.835957 -0.681396 -0.601986 -0.526842 -0.373229 0.00249406
v_subj(coke).13321 -0.537618 0.124244 -0.787294 -0.620311 -0.537078 -0.453282 -0.294107 0.00170903
v_subj(coke).13322 -0.132691 0.120955 -0.373582 -0.212934 -0.135703 -0.0520127 0.106469 0.00164423
v_subj(coke).13332 -0.154985 0.117702 -0.38697 -0.231234 -0.155284 -0.077276 0.0745538 0.00150829
v_subj(coke).13340 -0.616909 0.133357 -0.875259 -0.70737 -0.618911 -0.526365 -0.356376 0.00210942
v_subj(coke).13354 -0.0278574 0.125783 -0.277451 -0.112096 -0.0288249 0.0550303 0.217816 0.00193319
v_subj(coke).13374 0.104956 0.197141 -0.282286 -0.0263885 0.106181 0.23428 0.48986 0.00301978
v_subj(coke).13377 -0.200741 0.104579 -0.408379 -0.269902 -0.198255 -0.129906 0.00501685 0.00145045
v_subj(coke).13382 -0.771083 0.158141 -1.0742 -0.878404 -0.770593 -0.664305 -0.460611 0.00265208
v_subj(coke).13409 -0.774297 0.131442 -1.03635 -0.863344 -0.772022 -0.68288 -0.523224 0.00283765
v_subj(coke).13445 0.0596448 0.136738 -0.205336 -0.0316082 0.0603084 0.15417 0.327717 0.00213833
v_subj(coke).13450 -0.936129 0.167877 -1.26832 -1.04867 -0.938457 -0.820848 -0.6069 0.00317711
v_subj(coke).13456 -0.453481 0.109849 -0.665163 -0.525863 -0.456379 -0.379137 -0.239864 0.00160048
v_subj(coke).13459 0.306488 0.143104 0.0323155 0.211076 0.304343 0.402713 0.588436 0.00196437
v_subj(coke).13479 -0.287058 0.123409 -0.534423 -0.370907 -0.285346 -0.202543 -0.0485594 0.00206831
v_subj(coke).13482 -0.361323 0.124902 -0.601532 -0.448493 -0.361029 -0.275569 -0.11756 0.00179095
v_subj(coke).13483 -0.347871 0.149176 -0.641499 -0.447723 -0.345782 -0.24884 -0.0554707 0.00231317
v_subj(coke).13484 -0.566391 0.113262 -0.781633 -0.643631 -0.567663 -0.490014 -0.339284 0.00197279
v_subj(coke).13485 0.536247 0.137673 0.259951 0.444722 0.534488 0.628944 0.802747 0.00202616
v_subj(coke).13491 -0.608242 0.121 -0.846246 -0.6874 -0.608468 -0.525245 -0.36944 0.00219699
v_subj(coke).13514 -1.14465 0.176689 -1.49291 -1.26354 -1.14369 -1.02457 -0.802707 0.00341336
v_subj(coke).13523 -0.36825 0.142138 -0.644584 -0.465858 -0.371972 -0.273055 -0.0848351 0.00225404
v_subj(coke).13566 0.00534698 0.108957 -0.20779 -0.0674409 0.00677759 0.0788742 0.220026 0.00135384
v_subj(coke).13572 -1.06916 0.194141 -1.4662 -1.1966 -1.06766 -0.938192 -0.693564 0.00346092
v_subj(coke).13574 0.390071 0.143945 0.109082 0.293831 0.391076 0.487314 0.673545 0.00238103
v_subj(coke).13608 -0.199378 0.129205 -0.450771 -0.287882 -0.200147 -0.112537 0.0561296 0.00187001
v_subj(coke).13619 -0.285761 0.154509 -0.579593 -0.391592 -0.289115 -0.180741 0.0164214 0.00236669
v_subj(coke).13624 -0.670148 0.140463 -0.940897 -0.765034 -0.670531 -0.574849 -0.400301 0.00247331
v_subj(coke).13636 -0.249738 0.133292 -0.511793 -0.339762 -0.249584 -0.160633 0.00911155 0.00187359
v_subj(coke).13669 -0.355003 0.182816 -0.70489 -0.480235 -0.358839 -0.231289 0.0022418 0.00272717
v_subj(coke).13735 0.437645 0.130167 0.183229 0.34941 0.436479 0.525087 0.693731 0.00173271
v_subj(coke).13762 -0.795633 0.144193 -1.07753 -0.891333 -0.796487 -0.701506 -0.510119 0.00249204
v_subj(coke).13770 -0.0781442 0.122717 -0.315967 -0.161743 -0.0775027 0.00199695 0.169291 0.00186141
v_subj(coke).13808 0.129533 0.121381 -0.103774 0.0469368 0.129969 0.212664 0.364762 0.00165456
v_subj(coke).13958 0.259539 0.0918478 0.0788691 0.200205 0.260171 0.320938 0.439852 0.0013888
v_subj(coke).14004 0.427172 0.121983 0.192394 0.346414 0.426457 0.507518 0.666432 0.0019428
v_subj(coke).14014 0.127038 0.133815 -0.142956 0.0341465 0.128719 0.217578 0.385471 0.00186323
v_subj(coke).14020 0.0283513 0.122387 -0.209722 -0.0551643 0.027401 0.110162 0.265377 0.00189606
v_subj(coke).14027 -0.290025 0.151203 -0.587222 -0.39155 -0.289741 -0.187086 0.00722153 0.00251944
v_subj(neutral).12933 -0.561474 0.12494 -0.809637 -0.644965 -0.564613 -0.4766 -0.315495 0.00201635
v_subj(neutral).12945 -0.871718 0.156911 -1.17652 -0.976033 -0.877643 -0.763827 -0.55935 0.0025085
v_subj(neutral).13067 -0.420179 0.129949 -0.678956 -0.508351 -0.419809 -0.332699 -0.161748 0.00197617
v_subj(neutral).13076 -0.902869 0.158795 -1.22122 -1.01003 -0.901102 -0.794583 -0.592556 0.0029388
v_subj(neutral).13086 -1.03704 0.185052 -1.4054 -1.16188 -1.03462 -0.909756 -0.672308 0.00301331
v_subj(neutral).13110 -0.500366 0.156958 -0.808402 -0.605554 -0.501925 -0.39301 -0.188841 0.00216015
v_subj(neutral).13153 -0.547447 0.15263 -0.843013 -0.650901 -0.546781 -0.443444 -0.251309 0.00214148
v_subj(neutral).13202 0.285343 0.143071 0.00721817 0.19189 0.285001 0.383434 0.566718 0.00190691
v_subj(neutral).13206 -0.676298 0.158026 -0.977014 -0.784715 -0.677555 -0.569073 -0.366681 0.00247836
v_subj(neutral).13207 -0.449925 0.125435 -0.699815 -0.53109 -0.448647 -0.365358 -0.206067 0.00185123

v_subj(neutral).13213 0.107833 0.164939 -0.212438 -0.00324969 0.105088 0.216109 0.441828 0.0024603
v_subj(neutral).13217 -0.466516 0.155428 -0.775968 -0.568419 -0.465965 -0.360648 -0.161312 0.00226527
v_subj(neutral).13224 -1.00601 0.144291 -1.30144 -1.10147 -1.00619 -0.908801 -0.720058 0.00237487
v_subj(neutral).13227 -0.525235 0.129185 -0.775854 -0.615454 -0.526069 -0.435462 -0.267106 0.00197738
v_subj(neutral).13232 0.149342 0.122924 -0.0943879 0.0686281 0.150057 0.230071 0.393179 0.00168843
v_subj(neutral).13260 -0.511097 0.161455 -0.83001 -0.618156 -0.508475 -0.400494 -0.202353 0.00218844
v_subj(neutral).13266 -0.791193 0.133591 -1.05232 -0.879983 -0.790271 -0.703658 -0.53273 0.00344345
v_subj(neutral).13267 -0.339973 0.114405 -0.571161 -0.415567 -0.33803 -0.262956 -0.113815 0.00178204
v_subj(neutral).13306 -0.309049 0.107098 -0.519528 -0.381105 -0.308422 -0.235615 -0.099037 0.00160643
v_subj(neutral).13312 -0.776611 0.121585 -1.01468 -0.856947 -0.777943 -0.694355 -0.539487 0.00259566
v_subj(neutral).13321 -0.367466 0.124432 -0.609335 -0.453211 -0.369433 -0.281751 -0.12061 0.00204203
v_subj(neutral).13322 0.0838513 0.118569 -0.149985 0.00470321 0.0834186 0.163519 0.313747 0.00159746
v_subj(neutral).13332 -0.297271 0.122027 -0.537005 -0.381585 -0.297593 -0.213168 -0.0611727 0.00158928
v_subj(neutral).13340 -0.632962 0.146806 -0.92208 -0.732365 -0.630395 -0.532278 -0.347044 0.00225412
v_subj(neutral).13354 -0.227005 0.11425 -0.448602 -0.304214 -0.228392 -0.149649 0.000866927 0.0017259
v_subj(neutral).13374 -0.284853 0.191211 -0.651686 -0.415364 -0.285335 -0.157921 0.0856301 0.00288873
v_subj(neutral).13377 -0.0223412 0.117829 -0.251972 -0.0982193 -0.0233189 0.0565302 0.207082 0.00178458
v_subj(neutral).13382 -0.714829 0.148892 -1.00206 -0.815944 -0.716589 -0.613061 -0.424785 0.00272287
v_subj(neutral).13409 -0.801234 0.144877 -1.09412 -0.895727 -0.799853 -0.703835 -0.516198 0.00276262
v_subj(neutral).13445 0.179594 0.138562 -0.0899775 0.0844003 0.180762 0.272514 0.449794 0.00214697
v_subj(neutral).13450 -0.912669 0.168099 -1.24054 -1.02579 -0.914402 -0.799235 -0.575662 0.00257122
v_subj(neutral).13456 -0.404906 0.128241 -0.658077 -0.491633 -0.406389 -0.320867 -0.1477 0.00197944
v_subj(neutral).13459 0.145539 0.137293 -0.12192 0.051793 0.144283 0.239214 0.415107 0.00191895
v_subj(neutral).13479 0.00110384 0.115895 -0.227784 -0.0780959 0.00173544 0.0814728 0.218139 0.00172095
v_subj(neutral).13482 -0.432176 0.125333 -0.676365 -0.517367 -0.430667 -0.346171 -0.183869 0.00180335
v_subj(neutral).13483 -0.100709 0.14234 -0.380457 -0.196507 -0.0995807 -0.00592442 0.180252 0.00216412
v_subj(neutral).13484 -0.302718 0.101341 -0.501473 -0.371177 -0.302213 -0.233037 -0.106204 0.0016572
v_subj(neutral).13485 0.485851 0.13188 0.227668 0.395848 0.485368 0.573844 0.743063 0.00204888
v_subj(neutral).13491 -0.639632 0.119994 -0.875999 -0.719986 -0.641032 -0.558504 -0.403416 0.00207832
v_subj(neutral).13514 -1.00792 0.171536 -1.3471 -1.12165 -1.00815 -0.890492 -0.674559 0.00339445
v_subj(neutral).13523 -0.204482 0.121294 -0.439615 -0.285864 -0.2049 -0.122859 0.0312361 0.00176772
v_subj(neutral).13566 0.201076 0.110695 -0.0179444 0.127543 0.201124 0.27465 0.424511 0.00151692
v_subj(neutral).13572 -0.880228 0.190202 -1.24102 -1.00974 -0.882393 -0.756544 -0.504305 0.00332698
v_subj(neutral).13574 0.240883 0.119122 0.00877577 0.162576 0.239779 0.319997 0.478989 0.00192255
v_subj(neutral).13608 0.129878 0.131045 -0.123247 0.0404688 0.1274 0.217726 0.383801 0.00189206
v_subj(neutral).13619 -0.0922186 0.141244 -0.36542 -0.187214 -0.0913824 0.0018853 0.186726 0.00185292
v_subj(neutral).13624 -0.772437 0.147569 -1.06056 -0.872785 -0.773319 -0.673692 -0.480827 0.00275582
v_subj(neutral).13636 -0.194159 0.132511 -0.451377 -0.282429 -0.196267 -0.104311 0.0686509 0.00194918
v_subj(neutral).13669 -0.448668 0.184803 -0.804152 -0.577496 -0.446851 -0.324787 -0.0894657 0.00242724
v_subj(neutral).13735 0.29682 0.124725 0.0448101 0.213824 0.296358 0.37819 0.542056 0.00182989
v_subj(neutral).13762 -0.627843 0.146961 -0.917798 -0.724854 -0.624033 -0.530658 -0.339414 0.00262754
v_subj(neutral).13770 -0.0198154 0.11095 -0.235334 -0.092969 -0.0201789 0.0549947 0.20058 0.00161445
v_subj(neutral).13808 0.0777726 0.123499 -0.159065 -0.00624522 0.0783933 0.159409 0.324886 0.00192371
v_subj(neutral).13958 0.204226 0.0886282 0.0313891 0.142652 0.204501 0.265365 0.373941 0.00137937
v_subj(neutral).14004 0.345897 0.119324 0.114164 0.266868 0.346182 0.427124 0.573428 0.00168818
v_subj(neutral).14014 0.211985 0.12402 -0.0318028 0.130282 0.211916 0.295026 0.458387 0.00174848
v_subj(neutral).14020 -0.0737852 0.120966 -0.316553 -0.154253 -0.0744565 0.00764339 0.165058 0.00166283
v_subj(neutral).14027 -0.196635 0.143973 -0.476705 -0.295522 -0.196788 -0.101066 0.090872 0.00213629
v_subj(shape).12933 -0.752542 0.131435 -1.01061 -0.841415 -0.749658 -0.662979 -0.495939 0.00203452
v_subj(shape).12945 -0.876871 0.165517 -1.20375 -0.984971 -0.877064 -0.766542 -0.547893 0.0024266

v_subj(shape).13067 -0.236644 0.118235 -0.470107 -0.316285 -0.2364 -0.155691 -0.00783024 0.00178994
v_subj(shape).13076 -0.844613 0.15124 -1.1466 -0.946967 -0.843921 -0.74217 -0.547804 0.00272485
v_subj(shape).13086 -0.977036 0.171836 -1.31636 -1.08908 -0.978222 -0.863776 -0.640528 0.00279384
v_subj(shape).13110 -0.590342 0.153983 -0.899041 -0.691855 -0.589929 -0.488992 -0.294356 0.00220735
v_subj(shape).13153 -0.523976 0.14296 -0.812182 -0.617711 -0.523633 -0.425343 -0.25381 0.00229398
v_subj(shape).13202 0.224207 0.135772 -0.0508926 0.131031 0.226579 0.317036 0.482833 0.00195069
v_subj(shape).13206 -0.466777 0.142564 -0.747349 -0.560876 -0.465243 -0.370254 -0.193708 0.00220693
v_subj(shape).13207 -0.404929 0.122523 -0.643547 -0.488906 -0.405759 -0.320033 -0.157724 0.00168936
v_subj(shape).13213 -0.118568 0.137781 -0.39267 -0.211327 -0.114773 -0.0233591 0.145125 0.00200161
v_subj(shape).13217 -0.487022 0.13132 -0.740657 -0.576803 -0.484441 -0.398393 -0.23018 0.00194626
v_subj(shape).13224 -1.00745 0.13314 -1.26745 -1.09713 -1.00621 -0.917533 -0.752576 0.00216283
v_subj(shape).13227 -0.48741 0.116323 -0.706223 -0.56739 -0.48683 -0.410237 -0.249693 0.00181021
v_subj(shape).13232 -0.0285781 0.124946 -0.271975 -0.111829 -0.0277758 0.0544766 0.214986 0.00187007
v_subj(shape).13260 -0.604208 0.157064 -0.905642 -0.713189 -0.607525 -0.499848 -0.297121 0.00227766
v_subj(shape).13266 -0.847617 0.130969 -1.1097 -0.936107 -0.846803 -0.760322 -0.591045 0.00329629
v_subj(shape).13267 -0.45755 0.124308 -0.701807 -0.541469 -0.457344 -0.373448 -0.211768 0.00200091
v_subj(shape).13306 -0.521177 0.108704 -0.735302 -0.593935 -0.522467 -0.448679 -0.306962 0.00189347
v_subj(shape).13312 -0.748609 0.11999 -0.982093 -0.828478 -0.748083 -0.669692 -0.514256 0.00275096
v_subj(shape).13321 -0.423252 0.116046 -0.650605 -0.502603 -0.423395 -0.343514 -0.195516 0.00178934
v_subj(shape).13322 -0.0660581 0.103156 -0.270436 -0.137137 -0.0638335 0.00267493 0.1325 0.00161476
v_subj(shape).13332 -0.390924 0.12045 -0.621935 -0.471405 -0.393641 -0.311051 -0.15557 0.00177325
v_subj(shape).13340 -0.660316 0.131004 -0.912053 -0.748067 -0.660869 -0.572862 -0.400524 0.00226952
v_subj(shape).13354 -0.209254 0.115784 -0.435919 -0.289108 -0.209893 -0.129005 0.0170769 0.00168215
v_subj(shape).13374 -0.273554 0.182232 -0.632062 -0.396444 -0.276286 -0.156491 0.101528 0.00259055
v_subj(shape).13377 -0.409057 0.11169 -0.627862 -0.483822 -0.406912 -0.332352 -0.191549 0.00157586
v_subj(shape).13382 -1.0344 0.171108 -1.37602 -1.15023 -1.03514 -0.91784 -0.695841 0.00305862
v_subj(shape).13409 -0.830161 0.136741 -1.09641 -0.921676 -0.827149 -0.738109 -0.567163 0.00264882
v_subj(shape).13445 0.201347 0.138453 -0.0740715 0.108956 0.202469 0.292443 0.476186 0.00196199
v_subj(shape).13450 -0.660378 0.165151 -0.989868 -0.769439 -0.660173 -0.548012 -0.343806 0.00265821
v_subj(shape).13456 -0.405506 0.123598 -0.651754 -0.488471 -0.405293 -0.322717 -0.165224 0.00159773
v_subj(shape).13459 0.362417 0.137559 0.0935861 0.268709 0.363658 0.454979 0.62883 0.00199715
v_subj(shape).13479 -0.193511 0.119397 -0.425268 -0.276289 -0.192869 -0.11202 0.0328042 0.00158708
v_subj(shape).13482 -0.296874 0.121845 -0.542758 -0.380934 -0.295976 -0.21717 -0.0514877 0.00173233
v_subj(shape).13483 -0.266644 0.146982 -0.560675 -0.363982 -0.266392 -0.171233 0.0199275 0.00190771
v_subj(shape).13484 -0.516161 0.112282 -0.734766 -0.58772 -0.516516 -0.44291 -0.288539 0.00178857
v_subj(shape).13485 -0.275778 0.121712 -0.512041 -0.358054 -0.275199 -0.194133 -0.0332703 0.00166843
v_subj(shape).13491 -0.666615 0.12284 -0.912431 -0.747494 -0.666451 -0.584429 -0.432361 0.00175485
v_subj(shape).13514 -1.02888 0.16229 -1.3522 -1.13949 -1.02711 -0.917469 -0.709867 0.00331721
v_subj(shape).13523 -0.20233 0.132655 -0.465932 -0.289717 -0.201315 -0.11461 0.059457 0.00196131
v_subj(shape).13566 0.163025 0.107523 -0.0499424 0.0917459 0.164077 0.235695 0.371926 0.00160547
v_subj(shape).13572 -1.18626 0.200644 -1.57444 -1.32359 -1.18725 -1.05209 -0.789812 0.00366773
v_subj(shape).13574 0.122181 0.115812 -0.103593 0.0450652 0.123656 0.19788 0.358788 0.00177459
v_subj(shape).13608 -0.424733 0.130974 -0.68141 -0.511184 -0.425832 -0.335565 -0.16362 0.00182267
v_subj(shape).13619 -0.177011 0.154009 -0.476911 -0.283397 -0.175516 -0.0740396 0.128988 0.00230242
v_subj(shape).13624 -0.789041 0.141963 -1.06362 -0.884502 -0.790278 -0.69349 -0.5057 0.00267949
v_subj(shape).13636 -0.422429 0.131836 -0.685748 -0.507918 -0.421831 -0.334137 -0.166828 0.0017822
v_subj(shape).13669 -0.473104 0.178489 -0.822353 -0.590251 -0.474332 -0.353562 -0.124864 0.00272834
v_subj(shape).13735 0.119971 0.117381 -0.114601 0.0411182 0.120519 0.198807 0.354029 0.00163848
v_subj(shape).13762 -0.784765 0.141556 -1.05304 -0.879727 -0.78371 -0.692151 -0.496042 0.00261587
v_subj(shape).13770 -0.030633 0.11687 -0.260282 -0.109802 -0.0299643 0.0455645 0.203333 0.0017623

v_subj(shape).13808 -0.0741621 0.111163 -0.290203 -0.148483 -0.0750481 0.00209727 0.142145 0.00165702
v_subj(shape).13958 0.27004 0.083034 0.107078 0.212916 0.268666 0.327735 0.433073 0.00123467
v_subj(shape).14004 0.107858 0.106305 -0.102756 0.036926 0.108532 0.178519 0.313843 0.00156171
v_subj(shape).14014 0.109515 0.127734 -0.135499 0.0231059 0.110944 0.196229 0.355346 0.00191374
v_subj(shape).14020 -0.270294 0.119854 -0.503111 -0.347281 -0.269856 -0.189053 -0.0402945 0.00163967
v_subj(shape).14027 -0.172474 0.155094 -0.470965 -0.276346 -0.17273 -0.0681502 0.13107 0.00231002
t 1.12399 0.0929494 0.952951 1.05985 1.11935 1.1844 1.32028 0.00190418
t_std 0.7454 0.0881754 0.59756 0.683032 0.735551 0.800046 0.935834 0.0018674
t_subj.12933 0.743574 0.100745 0.517897 0.685262 0.755176 0.817147 0.905587 0.00199268
t_subj.12945 1.73002 0.0581349 1.59597 1.6955 1.73877 1.77104 1.82412 0.00117813
t_subj.13067 0.67622 0.0724058 0.502105 0.634699 0.686931 0.728345 0.787681 0.00127566
t_subj.13076 1.33425 0.0826833 1.14994 1.28371 1.34472 1.39475 1.46778 0.00178907
t_subj.13086 2.12452 0.0520399 2.00286 2.0957 2.13109 2.16099 2.20773 0.00124633
t_subj.13110 1.73598 0.0610577 1.59526 1.70147 1.74488 1.77941 1.82874 0.00118765
t_subj.13153 2.14684 0.0755895 1.97763 2.10087 2.15588 2.20045 2.26959 0.00162299
t_subj.13202 0.589927 0.0684239 0.436214 0.549472 0.599559 0.639031 0.698939 0.00146218
t_subj.13206 2.3748 0.0693081 2.21691 2.33638 2.38387 2.42462 2.48336 0.00134219
t_subj.13207 0.719984 0.0687114 0.559322 0.681035 0.729873 0.76864 0.825892 0.00130736
t_subj.13213 2.2518 0.0553384 2.12491 2.21875 2.25947 2.29244 2.33875 0.0011262
t_subj.13217 0.959209 0.102824 0.722 0.898836 0.974479 1.0332 1.12025 0.00206012
t_subj.13224 2.75499 0.0302092 2.68974 2.73614 2.75748 2.77645 2.80619 0.000644785
t_subj.13227 0.63231 0.0927335 0.42387 0.575308 0.643512 0.699746 0.784144 0.00198195
t_subj.13232 0.697706 0.0741201 0.521974 0.655521 0.707167 0.751048 0.811101 0.00147509
t_subj.13260 1.51693 0.0655852 1.36768 1.4785 1.52559 1.56456 1.61969 0.00126477
t_subj.13266 0.0531358 0.0287011 0.00857105 0.0308014 0.0497637 0.0713954 0.116251 0.000583028
t_subj.13267 0.565044 0.0967825 0.347008 0.508878 0.574647 0.633947 0.724027 0.00178433
t_subj.13306 0.552244 0.105945 0.312484 0.487098 0.564909 0.630014 0.723448 0.00194968
t_subj.13312 0.488567 0.112489 0.237551 0.420769 0.50022 0.568826 0.677498 0.00266744
t_subj.13321 0.647155 0.091807 0.433944 0.595968 0.660266 0.712172 0.788993 0.00178351
t_subj.13322 0.577407 0.101128 0.351812 0.516151 0.58849 0.65016 0.741406 0.00188022
t_subj.13332 0.710921 0.0799001 0.528691 0.662902 0.722837 0.767533 0.834834 0.00170676
t_subj.13340 0.711208 0.070013 0.552578 0.672424 0.720175 0.761268 0.821204 0.00141177
t_subj.13354 0.658452 0.0869791 0.456972 0.609527 0.669481 0.720373 0.795288 0.00167312
t_subj.13374 2.32828 0.0323243 2.25184 2.3099 2.33295 2.3515 2.37747 0.000719763
t_subj.13377 0.417696 0.103474 0.188356 0.353508 0.429226 0.493374 0.585484 0.00205929
t_subj.13382 1.86198 0.0682502 1.70745 1.82228 1.86994 1.91085 1.97173 0.00146903
t_subj.13409 0.733879 0.106057 0.498895 0.670118 0.744608 0.809761 0.908323 0.00294962
t_subj.13445 1.39708 0.0659289 1.2398 1.36052 1.40778 1.44314 1.49679 0.00121077
t_subj.13450 2.21344 0.0653365 2.06609 2.17572 2.2217 2.2605 2.31436 0.00141931
t_subj.13456 0.767064 0.105953 0.515173 0.708117 0.78185 0.842942 0.924526 0.0022128
t_subj.13459 1.08642 0.0630407 0.932519 1.05275 1.09677 1.13054 1.18256 0.00132866
t_subj.13479 0.760002 0.108466 0.515574 0.697919 0.775018 0.839174 0.930029 0.0019323
t_subj.13482 0.477971 0.109361 0.236146 0.41144 0.486742 0.557595 0.664071 0.00209612
t_subj.13483 1.82286 0.0726537 1.65247 1.7828 1.83306 1.87431 1.93499 0.0015852
t_subj.13484 0.670145 0.116633 0.411824 0.599597 0.680213 0.75682 0.858279 0.00218201
t_subj.13485 0.882243 0.0768829 0.697589 0.839401 0.895047 0.937732 0.996931 0.00123805
t_subj.13491 0.773784 0.0916657 0.571408 0.7182 0.78324 0.839343 0.923689 0.00166896
t_subj.13514 2.83259 0.0746648 2.65707 2.79102 2.84291 2.88618 2.94839 0.00202434
t_subj.13523 1.15809 0.0987631 0.920929 1.10185 1.1738 1.22898 1.30439 0.00179399
t_subj.13566 0.593443 0.104968 0.357838 0.530461 0.606464 0.668853 0.759307 0.00193689

```
t_subj.13572 2.73485 0.0508746 2.61995 2.70474 2.74145 2.77178 2.81512 0.0013379
t_subj.13574 0.408086 0.104297 0.182857 0.339675 0.416691 0.484228 0.586996 0.00200781
t_subj.13608 0.745111 0.0639679 0.600044 0.707964 0.752987 0.791955 0.842998 0.00125452
t_subj.13619 2.89423 0.0556514 2.76149 2.86329 2.90157 2.93364 2.98168 0.00115696
t_subj.13624 1.53868 0.0711875 1.37507 1.49744 1.54835 1.59008 1.64822 0.00145608
t_subj.13636 0.739326 0.0662745 0.583885 0.701499 0.747441 0.786794 0.842854 0.00123352
t_subj.13669 2.17837 0.0336904 2.09762 2.15993 2.18348 2.2029 2.22913 0.000649462
t_subj.13735 0.735966 0.065944 0.58723 0.696968 0.744883 0.784338 0.840805 0.00115339
t_subj.13762 0.79814 0.0769467 0.625527 0.752083 0.804629 0.8537 0.92612 0.00174347
t_subj.13770 0.643927 0.0811439 0.46151 0.595253 0.653121 0.702572 0.776387 0.00141499
t_subj.13808 0.703387 0.072857 0.533225 0.66127 0.713573 0.755997 0.817282 0.00135311
t_subj.13958 0.644164 0.0575273 0.517057 0.607775 0.649689 0.685198 0.740783 0.00111203
t_subj.14004 1.00627 0.0806832 0.827366 0.959531 1.01779 1.06441 1.13211 0.00160109
t_subj.14014 0.523335 0.0881973 0.318927 0.470929 0.531439 0.586156 0.672975 0.00233577
t_subj.14020 0.481492 0.0809907 0.29767 0.432468 0.489811 0.539412 0.61333 0.00164922
t_subj.14027 2.89183 0.0561988 2.75926 2.8608 2.89916 2.93085 2.98011 0.00109265
DIC: 17317.836397
deviance: 17060.069395
pD: 257.767002


In [10]: model1.plot_posteriors(['a', 'a_var'])
    ...: model1.plot_posteriors(['v', 'v_var'])
Plotting a(0)
Plotting a(1)
Plotting v(coke)
Plotting v(neutral)
Plotting v(shape)
/home/sdl/anaconda2/lib/python2.7/site-packages/matplotlib/figure.py:1999:
```

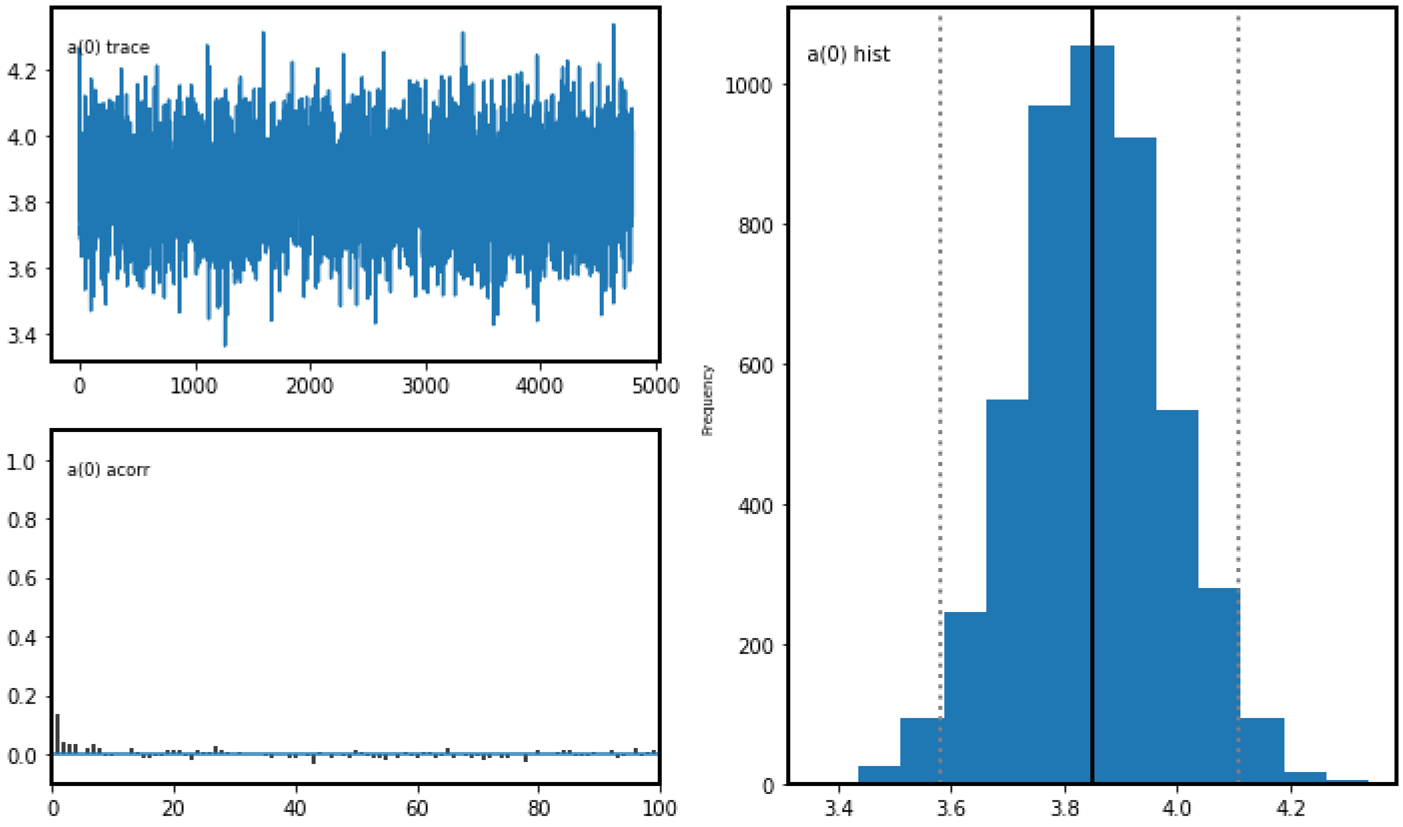

a(0) trace
4.2
4.0
3.8
3.6
3.4
0
1000
2000
3000
4000
5000
a(0) acorr
1.0
0.8
0.6
0.4
0.2
0.0
0
20
40
60
80
100
a(0) hist
Frequency
1000
800
600
400
200
0
3.4
3.6
3.8
4.0
4.2

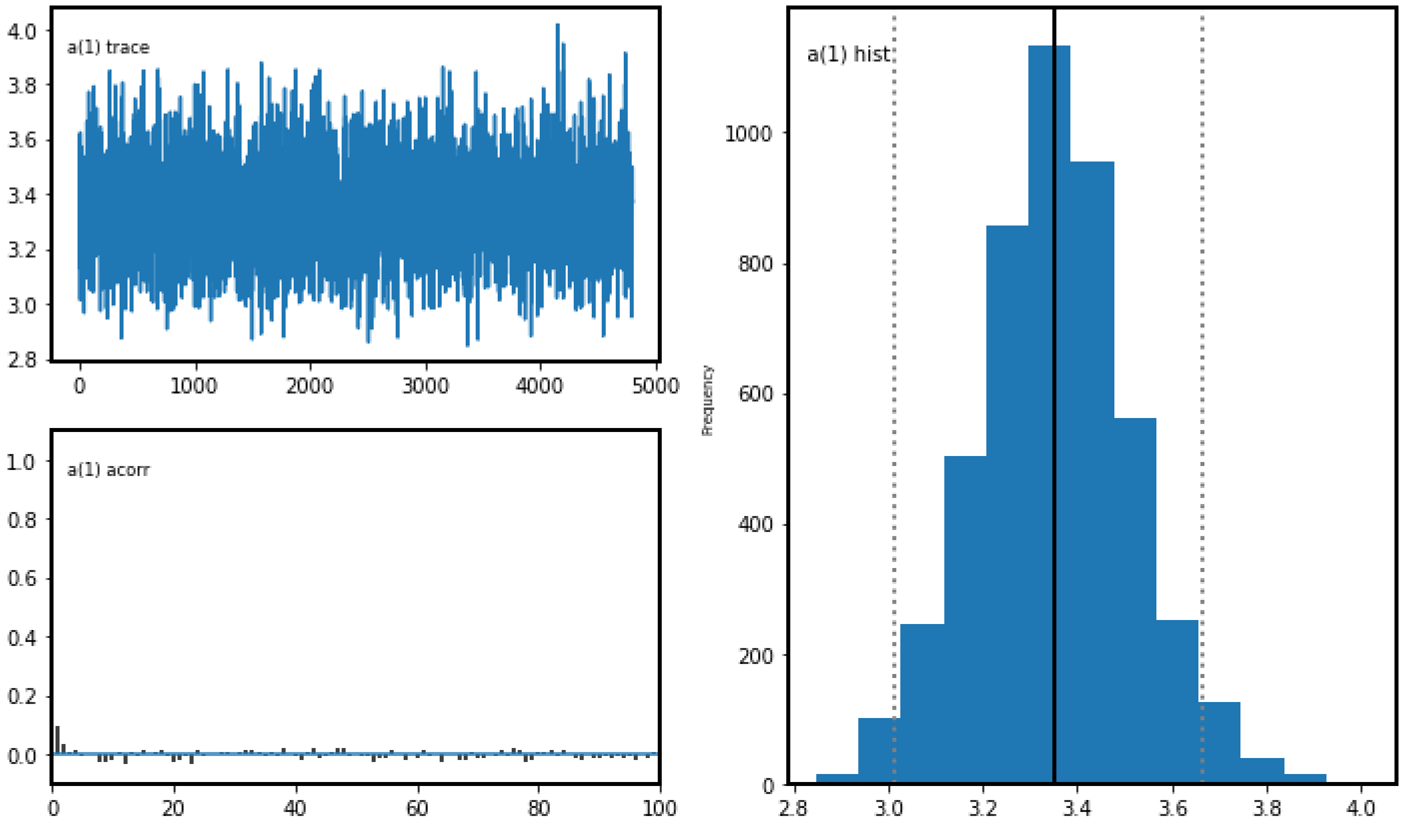

a(1) trace
a(1) acorr
a(1) hist
Frequency

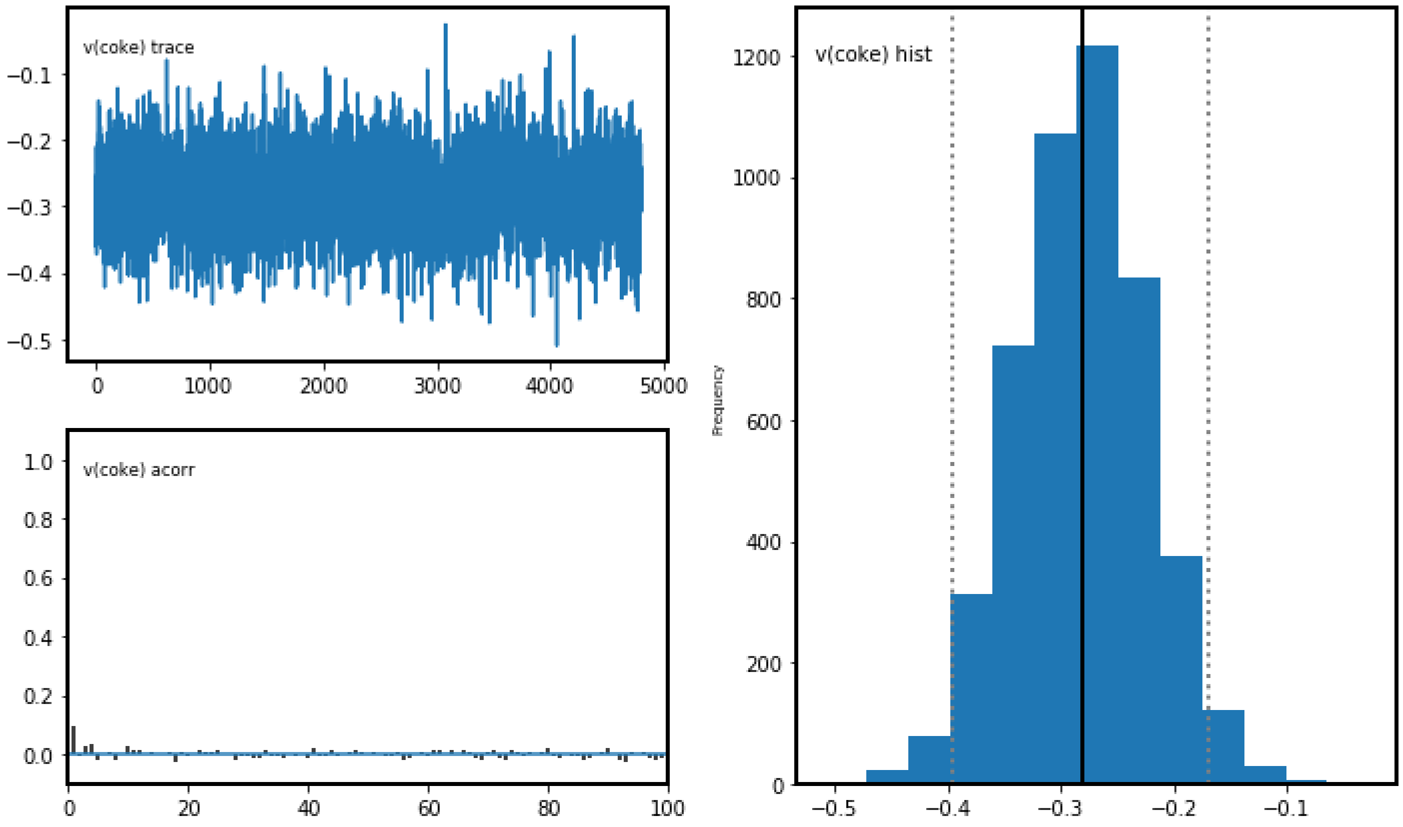
v(coke) trace
−0.1
−0.2
−0.3
−0.4
−0.5
0
1000
2000
3000
4000
5000
v(coke) acorr
1.0
0.8
0.6
0.4
0.2
0.0
0
20
40
60
80
100
v(coke) hist
Frequency
1200
1000
800
600
400
200
0
−0.5
−0.4
−0.3
−0.2
−0.1

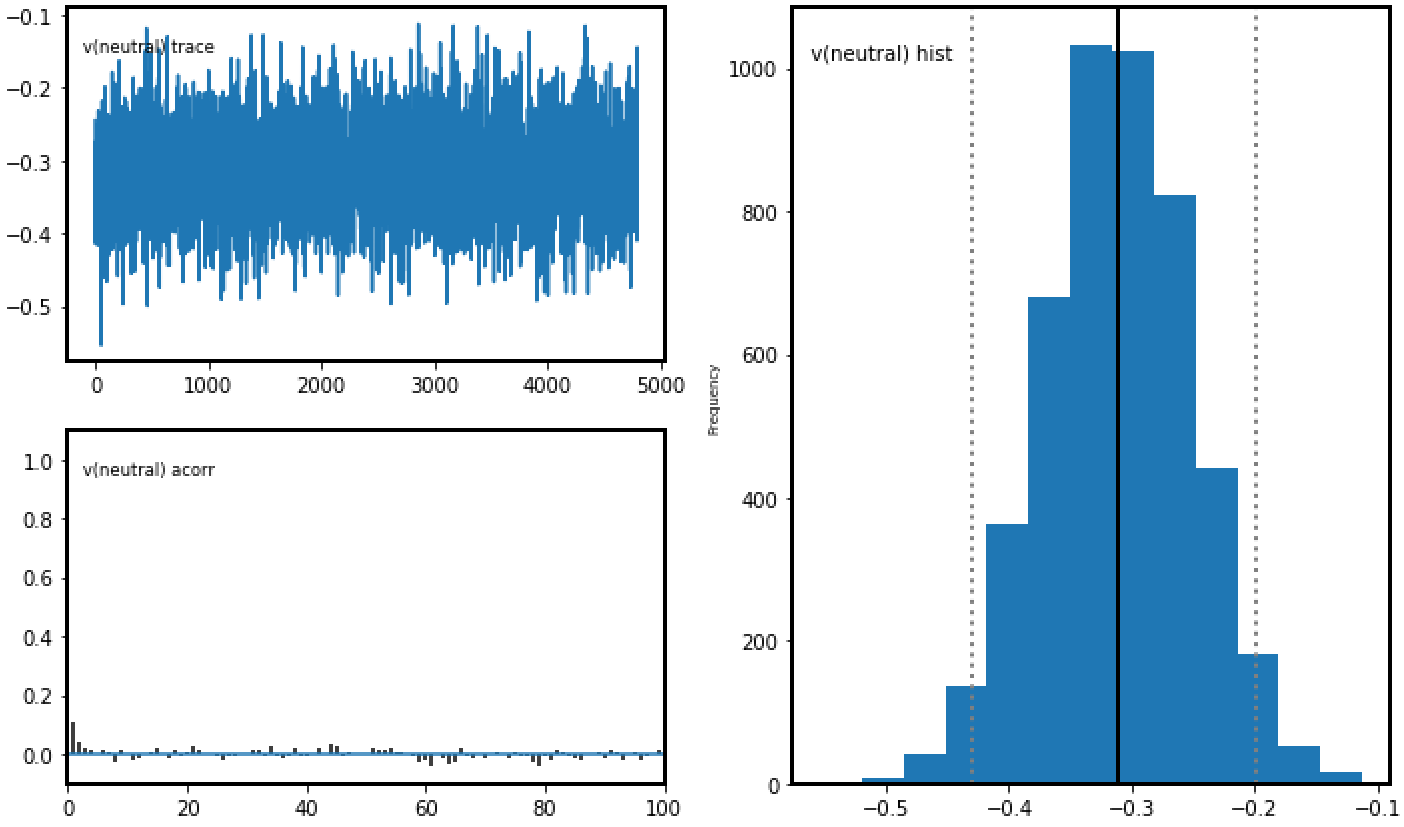
v(neutral) trace
v(neutral) acorr
v(neutral) hist
Frequency

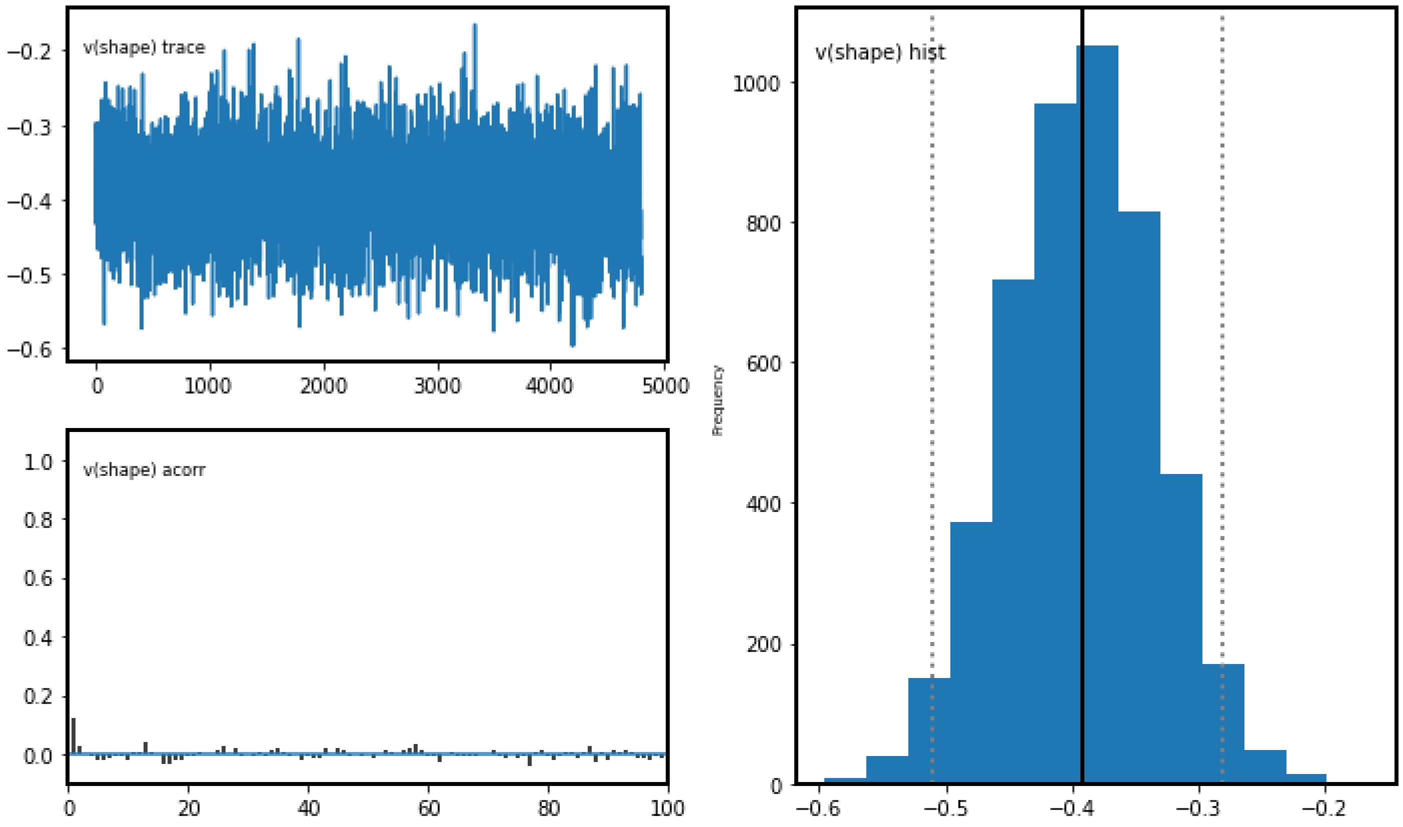
v(shape) trace
−0.2
−0.3
−0.4
−0.5
−0.6
0
1000
2000
3000
4000
5000
v(shape) acorr
1.0
0.8
0.6
0.4
0.2
0.0
0
20
40
60
80
100
v(shape) hist
Frequency
1000
800
600
400
200
0
−0.6
−0.5
−0.4
−0.3
−0.2
