# Supplementary material for: Measures of possible allostatic load in comorbid cocaine and alcohol use disorder: Brain white matter integrity, telomere length, and anti-saccade performance
Source: PLoS One. 2019 Jan 9;14(1):e0199729. doi: 10.1371/journal.pone.0199729 (PMC6326479; doi:10.1371/journal.pone.0199729)
Supplement: S3 File — Code, outcome parameters, and graphical information for the penalized regression using the elastic net via the R packages glmnet and selectiveInference. (PDF) [file pone.0199729.s003.pdf]

S3 File. Code, outcome parameters, and graphical information for the penalized regression using the elastic net via the R packages glmnet and selectiveInference.

```
# create matrix from x variables (predictors) and standardize
  x_matrix_tmp <- data.frame(data1$age, data1$v_cn_diff, data1$telo, data1$coke_yrs, data1$alc_yrs)
# subset the dataframe
  x_matrix <- data.matrix(x_matrix_tmp) # convert subsetted data frame to matrix
  xdata.z <- scale(x_matrix, center=T, scale=T)

# create vector for y variable (dti, dependent) and standardize
  y_vector <- as.vector(data1$rd_coke)
  ydata.z <- scale(y_vector, center=T, scale=T)

# run elastic net (lasso/ridge special case anchors) with LOOCV as per Finch and Finch article
  glmnet1.cv <- cv.glmnet(xdata.z,ydata.z, standardize = FALSE, type.measure="mse", nfolds=22)

# obtain lambda with min MSE
 glmnet1.cv$lambda.min
[1] 0.01899326

# obtain coeficients from model with optimal lambda value
 coef(glmnet1.cv,s = "lambda.min")

                 1
(Intercept)      -8.245236e-16
data1.age        3.754791e-01
data1.et_diff    3.279492e-01
data1.telo       -3.307578e-01
data1.coke_yrs   6.068837e-01
data1.alc_yrs    7.078269e-02

  # obtain r-squared from best fitted model
  r2 <- glmnet1.cv$glmnet.fit$dev.ratio[which(glmnet1.cv$glmnet.fit$lambda == glmnet1.cv$lambda.min)]
  r2
[1] 0.78524

  # examine relationship between coefficient values and model fit (as R-squared)
   glmnet2.z <- glmnet(xdata.z,ydata.z,alpha=1, standardize=FALSE, nlambda=100)
   plot(glmnet2.z, xvar="dev", label=TRUE, ylab=c("Regression Coefficient"), xlab=c("R Squared"))
```

```
# use selectiveinference package for covariance test estimate
# of the null hypothesis = no relationship with the dependent variable

 lasso.sigma <- estimateSigma(xdata.z,ydata.z, intercept=FALSE, standardize=FALSE)
 lasso.beta <- coef(glmnet2.z, glmnet1.cv$lambda.min )[-1]
 lasso.inference <- fixedLassoInf(xdata.z,ydata.z, lasso.beta, glmnet1.cv$lambda.min, sigma=lasso.sigma$sigmahat)

Call:
fixedLassoInf(x = xdata.z, y = ydata.z, beta = lasso.beta, lambda = glmnet1.cv$lambda.min,
    sigma = lasso.sigma$sigmahat)

Standard deviation of noise (specified or estimated) sigma = 0.546

Testing results at lambda = 0.019, with alpha = 0.100

 Var   Coef Z-score P-value LowCI   UpCI   LowTailArea UpTailArea
   1  0.375   2.862  0.004    0.148   0.595      0.048      0.050
   2  0.327   2.453  0.014    0.088   0.547      0.049      0.049
   3 -0.330  -2.363  0.047   -0.560  -0.004      0.049      0.049
   4  0.606   3.884  0.000    0.352   1.048      0.050      0.049
   5  0.075   0.540  0.592   -0.721   0.280      0.050      0.049

 Var labels: 1. data1.age  2. data1.et_diff   3. data1.telo  4. data1.coke_yrs  5. data1.alc_yrs

 sink() # write output
```

```
# examine fit parameters (variance) across the range of lambda from glmnet fit
> print(glmnet1.cv$glmnet.fit)

Call:  glmnet(x = xdata.z, y = ydata.z, standardize = FALSE)

     Df    %Dev   Lambda
 [1,]  0 0.00000 0.698600
 [2,]  1 0.09095 0.636600
 [3,]  1 0.16650 0.580000
 [4,]  1 0.22920 0.528500
 [5,]  1 0.28120 0.481500
 [6,]  1 0.32440 0.438800
 [7,]  1 0.36030 0.399800
 [8,]  1 0.39010 0.364300
```

[9,]	2	0.42360	0.331900
[10,]	2	0.45490	0.302400
[11,]	2	0.48090	0.275600
[12,]	2	0.50250	0.251100
[13,]	3	0.52200	0.228800
[14,]	4	0.56220	0.208500
[15,]	4	0.59970	0.189900
[16,]	4	0.63080	0.173100
[17,]	4	0.65670	0.157700
[18,]	4	0.67810	0.143700
[19,]	4	0.69600	0.130900
[20,]	4	0.71080	0.119300
[21,]	4	0.72300	0.108700
[22,]	4	0.73320	0.099030
[23,]	4	0.74170	0.090230
[24,]	4	0.74870	0.082220
[25,]	5	0.75520	0.074910
[26,]	5	0.76060	0.068260
[27,]	5	0.76510	0.062190
[28,]	5	0.76890	0.056670
[29,]	5	0.77200	0.051630
[30,]	5	0.77460	0.047050
[31,]	5	0.77670	0.042870
[32,]	5	0.77850	0.039060
[33,]	5	0.78000	0.035590
[34,]	5	0.78120	0.032430
[35,]	5	0.78220	0.029550
[36,]	5	0.78310	0.026920
[37,]	5	0.78380	0.024530
[38,]	5	0.78440	0.022350
[39,]	5	0.78480	0.020370
[40,]	5	0.78520	0.018560
[41,]	5	0.78560	0.016910
[42,]	5	0.78590	0.015410
[43,]	5	0.78610	0.014040
[44,]	5	0.78630	0.012790
[45,]	5	0.78640	0.011650
[46,]	5	0.78660	0.010620
[47,]	5	0.78670	0.009675
[48,]	5	0.78680	0.008816
[49,]	5	0.78680	0.008033

```
[50,]  5 0.78690 0.007319
[51,]  5 0.78700 0.006669
[52,]  5 0.78700 0.006076
[53,]  5 0.78700 0.005537
[54,]  5 0.78710 0.005045
[55,]  5 0.78710 0.004597
[56,]  5 0.78710 0.004188
[57,]  5 0.78710 0.003816
[58,]  5 0.78710 0.003477
[59,]  5 0.78710 0.003168
[60,]  5 0.78720 0.002887
[61,]  5 0.78720 0.002630
[62,]  5 0.78720 0.002397


glmnet1.cv$lambda.min   # obtain lambda with min MSE
[1] 0.0190806

> # obtain r-squared from best fitted model
> r2 <- glmnet1.cv$glmnet.fit$dev.ratio[which(glmnet1.cv$glmnet.fit$lambda == glmnet1.cv$lambda.min)]
> r2
[1] 0.7852

> RMSE <- sqrt(glmnet1.cv$lambda.min)
> RMSE
[1] 0.130031
```

> plot(glmnet1.cv)

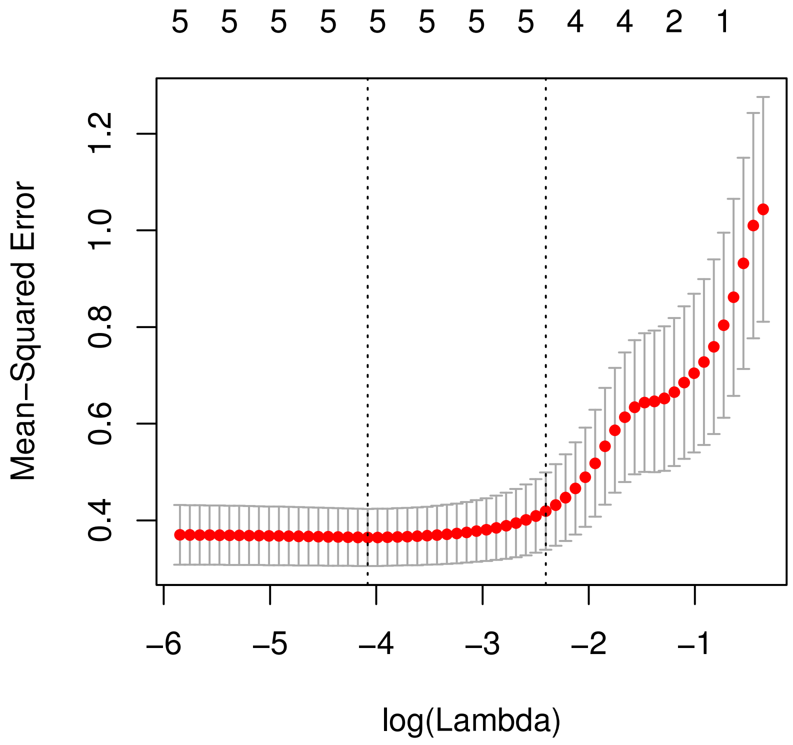

```
> plot(glmnet1.cv$glmnet.fit, xdata.z = "dev", label = TRUE)
```

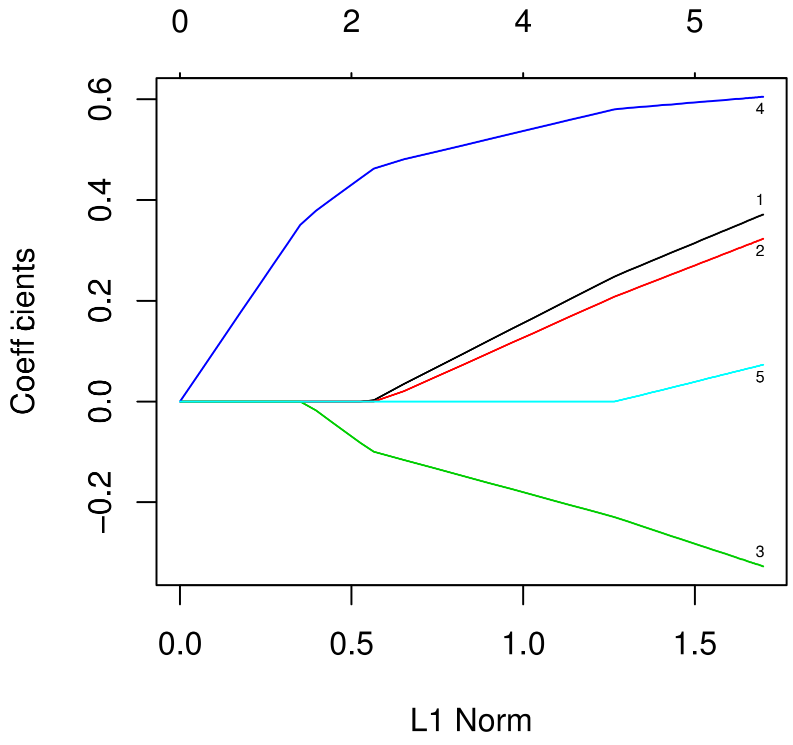

1. Age
2. Anti-saccade (drift rate difference: cocaine vs. non-drug)
3. Telomere length
4. Years of cocaine use
5. Years of alcohol use

```
> plot(glmnet2.z, xvar="dev", label=TRUE, ylab=c("Regression Coefficient (Lasso)"), xlab=c("R Squared"))
```

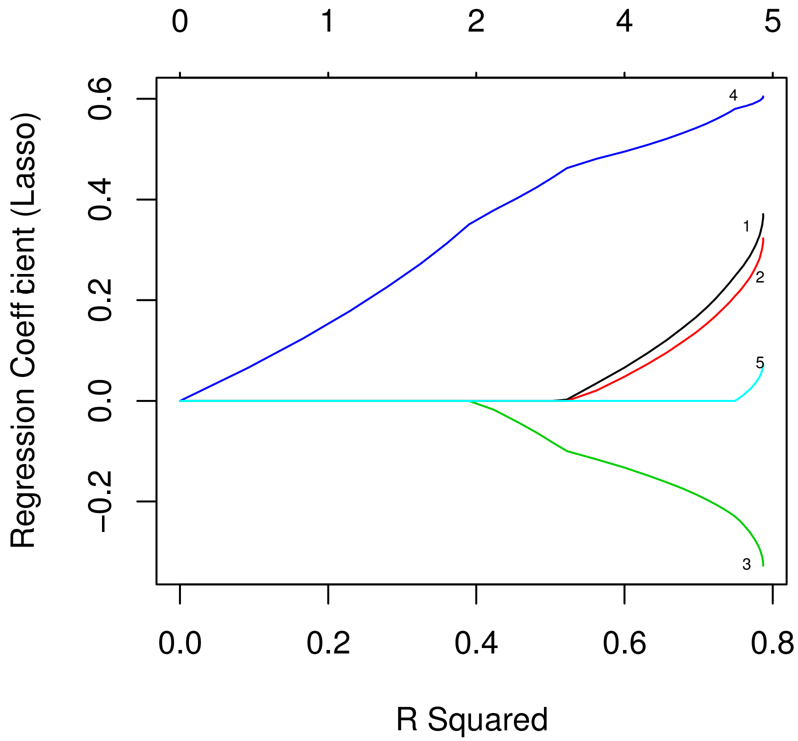

1. Age
2. Anti-saccade (drift rate difference: cocaine vs. non-drug)
3. Telomere length
4. Years of cocaine use
5. Years of alcohol use
