# Supplementary material for: Measures of possible allostatic load in comorbid cocaine and alcohol use disorder: Brain white matter integrity, telomere length, and anti-saccade performance
Source: PLoS One. 2019 Jan 9;14(1):e0199729. doi: 10.1371/journal.pone.0199729 (PMC6326479; doi:10.1371/journal.pone.0199729)
Supplement: S1 Table — Details for identified significant white matter (WM) clusters (number and percent) with location of tract label, for FA, MD, and RD values from the TBSS analyses regressing WM metrics and years of cocaine use, controlling for years of alcohol use, age, sex, and education. (PDF) [file pone.0199729.s004.pdf]

S1 Table. Details for identified significant white matter (WM) clusters (number and percent) with location of tract label, for FA, MD, and RD values from the TBSS analyses regressing WM metrics and years of cocaine use, controlling for years of alcohol use, age, sex, and education.

Tract Label	Total Tract Volume (voxels)	FA # sig voxels	FA % sig voxels	MD # sig voxels	MD % sig voxels	RD # sig voxels	RD % sig voxels	Tract Label Key
1	15644	0	0	0	0.00	0	0	Middle cerebellar peduncle
2	1500	0	0	0	0.00	0	0	Pontine crossing tract
3	8851	4536	51.25	868	9.81	3407	38.49	Genu of corpus callosum
4	13711	6628	48.34	452	3.30	6370	46.46	Body of corpus callosum
5	12729	2506	19.69	185	1.45	4578	35.97	Splenium of corpus callosum
6	659	0	0	0	0.00	0	0	Fornix (column and body)
7	1362	0	0	0	0.00	0	0	Corticospinal tract R
8	1370	0	0	0	0.00	0	0	Corticospinal tract L
9	690	0	0	0	0.00	0	0	Medial lemniscus R
10	699	0	0	0	0.00	0	0	Medial lemniscus L
11	968	0	0	0	0.00	0	0	Inf. cerebellar peduncle R
12	968	0	0	0	0.00	0	0	Inf. cerebellar peduncle L
13	992	0	0	0	0.00	0	0	Sup. cerebellar peduncle R
14	992	0	0	0	0.00	0	0	Sup. cerebellar peduncle L
15	2278	0	0	0	0.00	0	0	Cerebral peduncle R
16	2278	0	0	0	0.00	0	0	Cerebral peduncle L
17	3138	0	0	1156	36.84	1264	0	Ant. limb of internal capsule R
18	3018	0	0	0	0.00	362	11.99	Ant. limb of internal capsule L
19	3754	0	0	17	0.45	1431	38.12	Post. limb of internal capsule R

20	3752	0	0	0	0.00	0	0	Post. limb of internal capsule L
21	2515	2	0	51	2.03	446	17.73	Retrolenticular part of internal capsule R
22	2469	0	0	0	0.00	15	0.61	Retrolenticular part of internal capsule L
23	6849	1158	0	4217	61.57	4657	68.00	Ant. corona radiata R
24	6852	1906	0	0	0.00	4280	62.46	Ant. corona radiata L
25	7500	1336	0	3785	50.47	5372	71.63	Sup. corona radiata R
26	7508	2062	0	0	0.00	4067	54.17	Sup. corona radiata L
27	3728	1761	0	1996	53.54	2929	78.57	Post. corona radiata R
28	3714	376	0	0	0.00	1324	35.65	Post. corona radiata L
29	3972	255	0	2206	55.54	3320	83.59	Post. thalamic radiation (include optic radiation) R
30	3978	0	0	0	0.00	1816	45.65	Post. thalamic radiation (include optic radiation) L
31	2228	0	0	285	12.79	1545	69.34	Sagittal stratum (inf. longitidinal fasciculus and inf. fronto-occipital fasciculus) R
32	2231	0	0	0	0.00	0	0	Sagittal stratum (inf. longitidinal fasciculus and inf. fronto-occipital fasciculus) L
33	5611	0	0	337	6.01	194	3.46	External capsule R
34	5587	0	0	0	0.00	134	2.40	External capsule L
35	2342	37	0	4	0.17	339	14.47	Cingulum (cingulate gyrus) R
36	2751	69	0	0	0.00	61	2.22	Cingulum (cingulate gyrus) L
37	1236	0	0	0	0.00	0	0	Cingulum (hippocampus) R

38	1155	0	0	0	0.00	0	0	Cingulum (hippocampus) L
39	1124	0	0	0	0.00	1	0.09	Fornix (cres), Stria terminalis R
40	1125	0	0	0	0.00	0	0	Fornix (cres), Stria terminalis L
41	6607	285	4.31	2106	31.88	4273	64.67	Sup. longitudinal fasciculus R
42	6605	0	0.00	0	0.00	600	9.08	Sup. longitudinal fasciculus L
43	507	0	0.00	338	66.67	407	80.28	Sup. fronto-occipital fasciculus R (part of ant. internal capsule)
44	507	0	0.00	0	0.00	0	0	Sup. fronto-occipital fasciculus L (part of ant. internal capsule)
45	380	0	0.00	0	0.00	0	0	Uncinate fasciculus R
46	376	0	0.00	0	0.00	0	0	Uncinate fasciculus L
47	596	199	33.39	378	63.42	363	60.91	Tapetum R
48	600	0	0.00	0	0.00	186	31.00	Tapetum L

Ant. = Anterior

Sup. = Superior

Post. = Posterior
